# Gene-Activation Mechanisms in the Regression of Atherosclerosis, Elimination of Diabetes Type 2, and Prevention of Dementia

**DOI:** 10.2174/156652411795976556

**Published:** 2011-07

**Authors:** P.V Luoma

**Affiliations:** Institute of Biomedicine, Pharmacology, University of Helsinki, Finland

**Keywords:** Atherosclerosis, CYP, dementia, diabetes, endoplasmic reticulum, gene activation, HDL, LXR.

## Abstract

Atherosclerotic vascular disease, diabetes mellitus (DM) and dementia are major global health problems. Both endogenous and exogenous factors activate genes functioning in biological processes. This review article focuses on gene-activation mechanisms that regress atherosclerosis, eliminate DM type 2 (DM2), and prevent cognitive decline and dementia.

Gene-activating compounds upregulating functions of liver endoplasmic reticulum (ER) and affecting lipid and protein metabolism, increase ER size through membrane synthesis, and produce an antiatherogenic plasma lipoprotein profile. Numerous gene-activators regress atherosclerosis and reduce the occurrence of atherosclerotic disease. The gene-activators increase glucose disposal rate and insulin sensitivity and, by restoring normal glucose and insulin levels, remove metabolic syndrome and DM2. Patients with DM2 show an improvement of plasma lipoprotein profile and glucose tolerance together with increase in liver phospholipid (PL) and cytochrome (CYP) P450. The gene-activating compounds induce hepatic protein and PL synthesis, and upregulate enzymes including CYPs and glucokinase, nuclear receptors, apolipoproteins and ABC (ATP-binding cassette) transporters. They induce reparation of ER structures and eliminate consequences of ER stress. Healthy living habits activate mechanisms that maintain high levels of HDL and apolipoprotein AI, promote health, and prevent cognitive decline and dementia. Agonists of liver X receptor (LXR) reduce amyloid in brain plaques and improve cognitive performance in mouse models of Alzheimer`s disease.

The gene activation increases the capacity to withstand cellular stress and to repair cellular damage and increases life span. Life free of major health problems and in good cognitive health promotes well-being and living a long and active life.

## INTRODUCTION

Our survival depends upon the capacity to withstand cellular stress and repair molecular damage to maintain physiological functions**. **Both endogenous and exogenous factors influence the life maintaining activities. They influence the functions of genes that act as controllers of metabolic processes. Perturbations in lipid and protein metabolism are common in atherosclerotic cardiovascular diseases (CVDs) and metabolic disorders such as obesity, metabolic syndrome and type 2 diabetes (DM2) which are major health problems worldwide. Coronary heart disease (CHD) has been recognised as the most common cause of death in the world [1]. About nine out of ten patients with diagnosed DM in the U.S. have DM2 the prevalence of which has tripled in the last 30 years [2], and the prevalence of metabolic syndrome is rapidly increasing to epidemic proportions both in developed and developing countries [3]. The combination of obesity, elevated blood pressure and poor glucose tolerance often associates with elevated triglycerides and reduced HDL cholesterol (HDL-C) and together comprise the major components of the metabolic syndrome [4].

Clinical investigations performed in the 1970s revealed that drugs inducing protein synthesis and increasing liver proteins**, **in proportion to this effect produce plasma risk factor profile indicating a low probability of developing atherosclerotic disease [5, 6]. Studies in the 1980 including DM2 patients revealed that these gene-activating agents reduce both blood glucose and insulin [7, 8]. More recent studies have focused interest on gene-activation mechanisms affecting neuronal and cognitive function [9, 10]. A prospective study showed that removing modifiable risk factors of DM has a great reducing impact on the incidence of dementia, and suggested the elimination of DM as the principal target for health promotion programmes [11]. This review focuses on gene-activation mechanisms in the regression of atherosclerosis, elimination of DM2, and prevention of cognitive decline and dementia. 

## LIVER ENDOPLASMIC RETICULUM IN LIPID, PROTEIN AND GLUCOSE METABOLISM

The liver endoplasmic reticulum (ER) is the principal site for the synthesis and processing of lipids and proteins**, **and alterations in ER function influence plasma lipoprotein levels [12, 13]. Investigations performed in the 1960s revealed that induction of hepatic protein and lipid synthesis increases protein and phospholipid (PL) concentrations together with increase of ER membranes [14-16]. Phenobarbital (PB) has widely been used as a prototype for the evaluation of ER functions in protein, lipid, carbohydrate and xenobiotic metabolism. Studies performed in the 1970s and 1980s revealed that patients treated with PB-type gene-activating drugs show increase in liver protein and PL concentrations and P450 activity [5, 17] and decrease in hepatic triglycerides [18]. DM2 patients often have hepatic fatty degeneration together with increased triglyceride and reduced PL concentrations [19], and the gene-activating therapy reduces hepatic fat content as seen in light microscopy, and electron micrographs show reparation and increase in hepatocyte and ER membranes [7]. The therapy has similar effects also in obese mice with fatty liver [20]. The ER has a central role also in carbohydrate metabolism [21], and gene- activating compounds affect the fate of glucose. PB treatment reduces the activity of ER glucose-6-phosphatase [22] which is responsible for hepatic glucose production from glycogen breakdown or gluconeogenesis, and induces glucokinase [23], the major enzyme in the hepatic utilization of glucose. 

## HDL AND LDL RISK FACTORS AND ANTI-ATHEROGENIC MECHANISMS

HDL is known as the antiatherogenic lipoprotein acting in the reverse transport of cholesterol, and low density lipoprotein, LDL, as the atherogenic lipoprotein transporting cholesterol to tissues, including arteries. Apo AI and AII are major apolipoproteins of HDL and apo B the apolipoprotein of LDL. Two main subclasses, HDL2 and HDL3, can be separated in HDL density range. HDL2 is a large lipoprotein with high apo AI and PL content [24]. Phosphatidylcholine (PC) is the main PL (over 70%) of HDL [25], and the PC- and apo Al-rich HDL2 is known as the lipoprotein responsible for the antiatherogenic effect of HDL [26]. High HDL2-C level, HDL2-C/HDL3-C and apo AI/AII ratio, and low LDL-C level, and LDL-C/ HDL-C and apo B/apo AI ratio indicate a low risk of atherosclerotic disease. 

Sophisticated regulatory systems maintain cholesterol homeostasis (reviewed in [27-29]). They include cytochrome (CYP) P450-monooxygenases, physiological factors in the catabolism of cholesterol to oxysterols and bile acids and activation of cholesterol-lowering mechanisms [30, 31]. Oxysterols are endogenous ligands for liver X receptors (LXRα and LXRβ), and suppressors of hydroxyl-methylglutaryl CoA reductase (HMGCoAR), the rate-limiting enzyme in cholesterol synthesis, and their secretion from cells represents a form of cholesterol elimination. The LXRs mediate the expression of multiple genes in the regulation of cholesterol balance in the body, i.e. its cellular efflux, transport, excretion and absorption. LXRs upregulate ABC transporters including ABCA1, ABCG1, ABCG4, ABCG5 and ABCG8 that shuttle intracellular cholesterol for the efflux out of cells, and several apolipoproteins including apo AI and E. ABCA1 is a key playmaker in cholesterol efflux, and a major determinant of plasma HDL level [29]. An increased ABCA1 and ABCG1 expression stimulates cellular cholesterol efflux to lipid poor apo AI and HDL, respectively [28]. 

LDL particles accumulated in the arterial wall can undergo oxidative modification [32]. Oxidized LDL contains products of lipid peroxidation including lipid hydroperoxides and oxidized phospholipids which are key factors in the initiation and progression of atherosclerotic lesions [32, 33]. Apart from the key role in cholesterol efflux, LXR activation is important in protecting cells from the atherogenic effects of lipid peroxides and inflammation [34]. HDL, apo AI, and apo AI-mimetic peptides prevent LDL oxidation and decrease atherosclerotic lesions, and improve vascular reactivity in animal models and in humans [32]. Apo AI in HDL [35] and plasma glutathione peroxidase [36] reduce PC hydroperoxides, and an infusion of apo Al-PC discs raising low HDL is effective in restoring the endothelial function [37]. 

## EFFECTS OF GENE-ACTIVATING COMPOUNDS ON LIVER ER, RISK FACTORS, ATHERO-SCLEROSIS AND SURVIVAL

Healthy young nonobese subjects show high plasma HDL-C and HDL2–C and high HDL-C/ total cholesterol (T-C) and HDL2-C/HDL3-C ratio together with high plasma antipyrine (AP) clearance rate [38], which has been used as a measure of hepatic P450**-**activity *in vivo.* Functional crosstalk of common regulatory factors links lipid and xenobiotic metabolism and P450 activity [31, 39, 40]. A prospective, double-blind, placebo-controlled trial including nonepileptic subjects showed that phenytoin therapy raises plasma HDL-C, and particularly HDL2-C, and has no significant effect on HDL3-C, LDL-C, T-C and triglycerides [41]. 

**Fig. (1):**
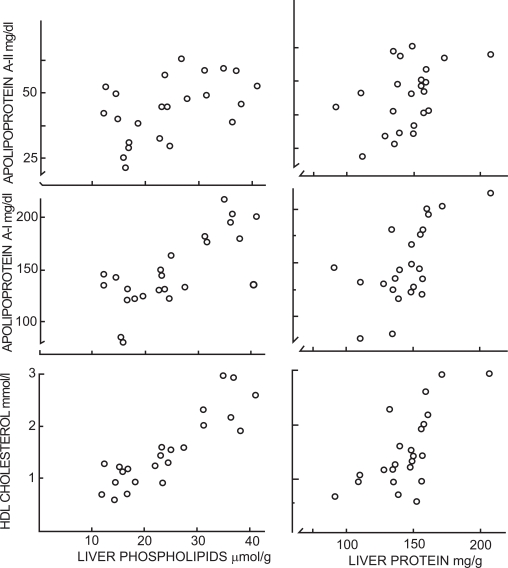
Relation of liver phospholipid and protein concentrations to plasma HDL cholesterol (r=0.878 and r=0.670, respectively), apolipoprotein A-I (r=0.812 and r=0.614) and A-II (r=0.433 and r=0.408) levels in 23 subjects. Reproduced from: Luoma PV, *et al*. Acta Med Scand., 1983; 214: 103-109 [17].

Plasma HDL-C and apo A-I raise with increasing protein, PL (Fig. **1**) [17], and P450 in the liver, and they are high in subjects undergoing gene-activating drug therapy [6, 42]. The subjects also show low LDL-C and LDL-C/HDL-C ratio [40] and high HDL2-C and HDL-C/ T-C ratio [38] together with high AP clearance rate. Plasma HDL-C, apo AI and HDL-C/ T-C ratio raise and triglycerides decrease with decreasing hepatic triglycerides [17], and fat content as determined microscopically [43]. These original studies linking gene activation, upregulation of hepatic ER functions, proteins, PL and P450 activity, with beneficial changes in key risk factors presented novel mecahanisms to prevent and treat atherosclerotic vascular disease [5, 31].

Hepatic CTP:phosphocholine cytidylyltransferase-α (CCTα), the regulatory enzyme for the synthesis of PC, is a key player in maintaining plasma HDL-C level [44]. Elevation of cellular cholesterol induces generation of oxysterols that upregulate CCTα and PC synthesis [45]. PC is the most important PL in reverse cholesterol transport because it is the essential cholesterol-binding component of lipoproteins and the acyl donor in the esterification of free cholesterol by lecithin:cholesterol acyltransferase (LCAT) [26]. In agreement with these studies, high PL content in HDL2 particles has been found to be an efficient driving factor for cholesterol removal from peripheral cells [46, 47], an intravenous infusion of apo AI/PC discs to stimulate reverse cholesterol transport in humans [48], and plasma HDL-PL levels to inversely correlate to the extent of atherosclerosis in coronary arteries [49]. 

Numerous xenobiotic compounds upregulate antiatherogenic mechanisms (reviewed in [50]). The compounds include drugs for dyslipidemias such as statins, niacin, fibrates, resins, as well as compounds for other purposes, including angiotensin converting enzyme (ACE) inhibitors, angiotensin receptor blockers, calcium channel blockers, glitazones and anticonvulsants, and alcohol. A number of xenobiotic gene-activators raise HDL2-C [50] and produce plasma lipoprotein pattern which is typical of low risk of atherosclerotic disease and exceptional longevity [51, 52], regress atherosclerosis, and reduce mortality and /or morbidity from CHD, cerebrovascular disease, and also death from any cause [50]. 

## EFFECTS OF GENE-ACTIVATORS ON LIVER ER AND GLUCOSE AND INSULIN

Clinical investigations performed in the 1980s evaluated the effects of gene-activating agents on glucose and insulin. The studies including DM2 patients revealed that the therapy induces plasma membrane reparation and increases the surface density in ER membranes of hepatocytes together with an elimination of liver fat, a decrease both in blood glucose and plasma IRI levels and enhancement of P450-mediated AP clearance [7]. These original observations linking upregulation of ER membranes and functions and lowering of glucose and IRI levels presented a novel approach for the prevention and treatment of DM2 [7]. Subsequent studies clarified, by using the euglycemic clamp technique, the effects of gene-activating compounds on glucose- insulin homeostasis both in healthy volunteers and patients with DM2 [8, 53]. The results revealed that a short-term PB treatment of healthy volunteers increases glucose disposal rate by approximately 30% and also the metabolic clearance rate of glucose, with concomitant enhancement of AP elimination [53]. PB therapy also reduced fasting IRI, while fasting blood glucose in healthy subjects remained unaltered. In contrast to PB effects, cimetidine, an inhibitor of P450, reduced glucose disposal rate and glucose metabolic clearance rate, and AP elimination [53]. 

**Fig. (2):**
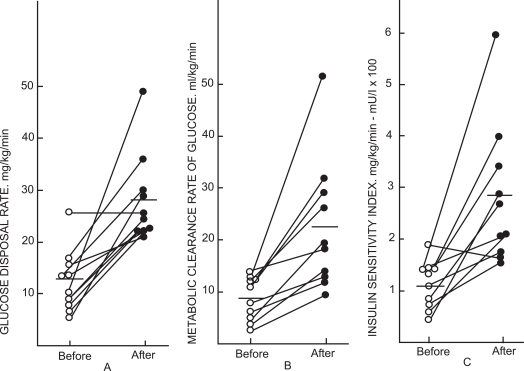
Change in glucose disposal rate (**A**), in metabolic clearance rate of glucose (**B**) and insulin sensitivity index (**C**) for individual type 2 diabetic subjects during glucose clamp studies performed using an insulin infusion rate of 1 mU/ kg before and after Phenobarbital therapy. Reproduced from: Lahtela JT, *et al*. Diabetes, 1985; 34: 911-6 [8], with permission from American Diabetes Association.

In subjects with liver disease glucose disposal rate varies proportionately to the amount of unaltered hepatic parenchyma as evaluated morphometrically, and to hepatic P450-mediated AP kinetics [54]. The glucose disposal rate was lowest among patients with fatty liver and those with liver cirrhosis. PB treatment of DM2 patients reduced both fasting blood glucose and IRI [8]. In addition, glucose disposal rate, metabolic clearance rate of glucose, and insulin sensitivity index, determined by using the euglycemic clamp technique, increased during therapy (Fig. **2**) [8]. 

Glucose has recently been identified as a natural LXR agonist [21]. It activates LXR at physiological concentrations expected in the liver and induces expression of LXR target genes with efficacy similar to that of oxysterol and determines its own fate [21]. Glucose binds and stimulates the transcriptional activity of LXR and coordinates hepatic lipid metabolism. Hepatic LXR activation suppresses gluconeogenic genes, and induces glucokinase in the liver that promotes hepatic glucose utilization [55]. 

## COGNITIVE IMPAIRMENT – MECHANISMS AND RISK FACTORS

Mild Cognitive Impairment (MCI) has been used to describe the transitional state between normal cognitive function and Alzheimer`s disease (AD). AD is the most common cause of dementia [56], the prevalence of which doubles every five years between 65 and 85 years of age [57]. The accumulation of amyloid β (Aβ) in the brain has been considered as the main culprit in the pathogenesis of AD, resulting in synapse disruption and neuronal destruction. Cognitive processes require gene expression modification to consolidate information, and transcriptional dysregulation could perturb neuronal function and cognitive performance [58].

Recent studies in the AD context have focused attention on LXRs [9, 59] and the so-called LXR-ABCA1- apo E axis [10, 60]. LXRs have an important function in lipid homeostasis in the brain, and the loss of the receptors leads to neuroregenerative processes [59]. ABCA1 has been shown to influence brain levels and lipidation of apo E in mice. A poor lipidation of apo E increases amyloid burden in mouse models of AD and, conversely, an overexpression of ABCA1 in the brain promotes apo E lipidation and reduces the formation of mature amyloid plaques [60]. LXR activating compounds reducing amyloid load also improve cognitive performance in mouse models of AD [61]. An activation of pregnane X receptor (PXR) may also improve cognitive function. Pregnenolone-16α-carbonitrile (PCN), a PXR agonist, has been shown to induce the expression of blood-brain barrier protein known as P-glycoprotein, and to reduce brain Aβ levels in a mouse model of AD [62]. This protein specifically mediates the efflux transport of Aβ from mouse brain capillaries into vascular space, a critical component of the Aβ brain efflux mechanism [69]. A recent study using a mouse model of AD and analyzing the effects of phenylbutyrate, revealed that the drug reduces AD linked tau protein and ameliorates cognitive deficit [58]. The drug is already approved for clinical use, and it may provide a novel approach for the treatment of AD [58]. 

Low HDL has been identified as a risk factor for deficit and decline in memory in midlife [63], and high HDL-C and apo AI concentrations correlate with cognitive function in advanced age [64]. Hyperinsulinemia associated with insulin resistance reduces insulin transport across the blood brain barrier, BBB, and subsequently lowers insulin levels and activity in the brain. The reduced brain insulin signaling associates with increased Aβ levels in mouse model of diabetes [65], and hyperinsulinemia increases risk of AD in man [56]. CYP enzymes could also influence the development of AD [66]. Polymorphism of CYP46, the cholesterol metabolizing enzyme in the brain, has been shown to influence brain Aβ- load, cerebrospinal fluid levels of Aβ peptides, and the risk of late-onset AD in man [67]. 

## DISTURBANCE AND RESTORATION OF ER FUNCTION AND METABOLIC DISEASE

Recent studies on the pathogenesis of metabolic diseases have clarified mechanisms that by disturbing ER homeostasis cause accumulation of unfolded or misfolded proteins and result in a state known as ER stress [68, 69]. During ER stress, these proteins are prevented from trafficking to their proper subcellular localizations and are usually rapidly regraded [70]. A disruption in the condition of this organelle affects the fate of lipids, proteins, glucose and insulin and associates with common ailments including atherosclerosis, DM, obesity and neurodegenerative disease [31, 70]. ER responds to the stress by triggering signalling cascade called unfolded protein response (UPR) for restoring the metabolic balance. A failure in this response can promote the disease process. The UPR maintains ER homeostasis by two connected mechanisms; by providing new ER-folding machinery and by providing ER expansion with increase in ER surface area and luminal space [71]. Gene-activating agents including P450-inducers increase ER size through membrane synthesis [31] that is an integral yet distinct part of the cellular program to overcome ER stress [71]. Chaperones are specialized proteins with a key role in cellular homeostasis by assisting in protein folding, assembly of the macromolecular complexes, protein transport and cellular signalling [70]. 

A dysregulation of ER function causing fatty regeneration in the liver, unfavorably affects lipoprotein metabolism, while an activation of ER functions increases ER membranes, reduces hepatic lipid deposition [7, 18], and has beneficial effects on lipoprotein risk factors [6]. X box-binding protein 1 (XBP-1) is a transcription factor which directs cells to construct more ER membranes for maintaining ER function [72]. An activation of the factor enhances CCTα activity and the synthesis of protein and PC, which is the principal PL of ER membranes and plasma lipoproteins [44]. Gene-activating compounds induce protein and PC synthesis and increase PC**-**rich HDL2 [50]. The PCs are essential cholesterophilic components of HDL2 in the reverse transport and elimination of cholesterol [26]. 

An accumulation of cholesterol and other lipids in macrophages can cause ER stress and eventually lead to the death of the cells [68]. Macrophage death in advanced atherosclerotic lesions could have deleterious consequences, such as exacerbated metabolic dysreglation and the rupture of vascular plaques. Phenylbutyrate, a chemical chaperone has been shown to mitigate macrophage ER stress and apoptosis in atherosclerotic lesions *in vivo*, indicating that the therapy improving ER chaperoning function can protect against the deleterious effects of toxic lipids in promoting atherosclerotic lesions [73]. 

Factors disturbing ER function affect the fate of glucose and promote hyperglycemia through insulin resistance, stimulation of hepatic glucose production and suppression of glucose disposal [8, 68], and gene-activating therapy can restore normal glucose-insulin homeostasis [7, 8]. Lipid, protein and glucose metabolism are linked and, correspondingly, DM2 patients show improvement of both glucose tolerance and plasma lipoprotein profile together with increase in liver PL and P450 [19]. An analysis of biopsies from obese subjects after weight loss and subsequently reduced adiposity after gastric bypass surgery showed reduced ER stress in liver and adipose tissue together with a 90 % decrease in liver triglyceride content and improvement of insulin sensitivity [74]. Tauroursodeoxycholic acid (TUDCA), a chemical chaperone used to treat cholelithiasis and cholestatic liver disease, has been shown to alleviate ER stress in obese and diabetic mouse models, normalize hyperglycemia, increase insulin sensitivity, resolve the fatty liver disease and enhance insulin action in liver, muscle and adipose tissues [75]. First studies testing TUDCA in obese subjects with insulin resistance have also resulted in promising results, an increase in hepatic and muscle insulin sensitivity [76]. 

## METABOLIC AND HEALTH EFFECTS OF LIFESTYLE FACTORS

Major lifestyle factors affecting health include poor dietary habits together with unhealthy composition of the diet with excess calories, physical inactivity, inadequate weight control, obesity, smoking, and excessive alcohol consumption. 

***Dietary factors*** have favorable metabolic effects. Weight control by a low calorie diet with adequate nutrition increases insulin sensitivity and reduces insulin secretion [77]. The effect of the diet on sirtuin 1 (SIRT1) could contribute to atheroprotection. The enzyme is an activator of LXR and cholesterol efflux *via *HDL formation. SIRT1 overepression could also induce hepatic CYP7A1, decrease hepatic cholesterol and *via *upregulation of the LDLR pathway reduce plasma LDL-C [78, 79]. Diet constituents such as soy proteins and alcohol upregulate apo AI gene and raise HDL-C [80], and vitamin B3 or niacin increases apo AI production [81]. Natural mechanisms modify the effects of unhealthy diet. A278C polymorphism in hepatic CYP7A1 gene affects the effect of a high-cholesterol diet on plasma HDL-C and cholesterol [82]. The increase in plasma cholesterol in the subjects with CC (Cytosine – Cytosine) genotype was mainly due to the larger increase in HDL-C. The activation of nuclear receptors could also modify the effect of the diet. Recent studies revealed that activation of constitutive androstane receptor (CAR) which is highly expressed in the liver, prevents diet-induced obesity, reduces hepatic steatosis, improves insulin sensitivity [83], and ameliorates diabetes and fatty liver disease in mice [84]. 

Dietary factors activating ER functions and stimulating cholesterol effluxing mechanisms, prevent and regress atherosclerosis. Moderate alcohol consumption protects from both atherosclerotic disease and dementia [85], and reduces CHD and all-cause mortality [31], and the Mediterranian-type of diet has multiple beneficial effects. The people on the diet have low blood pressure and fasting blood glucose and IRI levels, an antiatherogenic plasma risk factor profile [86], and low mortality rate from CHD, CVD, cancer, and also total mortality [50]. Adherence to the diet also associates with reduced risk of AD [87]. A healthy diet also reduces insulin secretion and increases insulin sensitivity and prevents the occurrence of metabolic syndrome [88], and also DM2 in people with impaired glucose tolerance [89], both of which impair cognitive performance [56].

***Regular physical activity*** has beneficial metabolic effects (reviewed in [50]). It upregulates receptors such as LXRα, PPARγ (peroxisome proliferator-activated receptor γ), scavenger receptor B1 (SRB1) and LDLR, enzymes such as CYPs, LCAT and paraoxonase 1, and transporters such as ABCA1, ABCG1 and apo AI. It also raises plasma apo AI, HDL-C and HDL2-C, and decreases LDL-C, cholesterol and triglycerides, and promotes cellular cholesterol efflux. Regular exercise improves oxygen uptake and weight control, lowers blood pressure, upregulates mechanisms protecting arteries from atherosclerosis and also decreases CHD, cardiovascular, cancer, and also total mortality [50]. Moderate exercise performed in midlife or late life also associates with later reduced odds of having MCI [90], and reduces the risk for AD [87]. In addition, physical activity reduces insulin resistance and postprandial hyperglycemia, improves glucose tolerance and prevents the development of DM2 in cases with impaired glucose tolerance [89]. 

## DISCUSSION

Both endogenous and exogenous factors including living habits activate functions of genes with favorable effects on major health problems. The gene-activators regress atherosclerosis, by normalization blood glucose – insulin homeostasis remove metabolic syndrome and DM2, and prevent cognitive decline and dementia. Morover, preclinical animal studies show that the compounds eliminate amyloid from brain plaques and improve cognitive performance. The compounds induce reparation and expansion of ER membranes and normalize or improve ER functions that maintain cellular metabolic homeostasis. They protect cells from the effects of inflammation and immune response and eliminate fat in cases with hepatic fatty degeneration. The gene-activation mechanisms correcting disturbed ER functions eliminate ER stress that links atherosclerosis, obesity, insulin action and DM2. 

Together with inducing protein synthesis and reparation of cellular structures, the gene-activators increase hepatic protein and PL and stimulate enzymes including CYPs and glucokinase. They also upregulate NRs, apolipoproteins and ABC transporters, and further produce antiatherogenic plasma lipoprotein profile and enhance glucose disposal rate and insulin sensitivity. Numerous gene-activators raise plasma HDL2 with high PC content [50], effective driving factor in key steps of the reverse cholesterol transport [26], and DM2 patients show improved glucose tolerance with increase in liver PL and P450 [19]. 

The liver has a key role in glycemic control. It produces more than 90% of endogenous glucose, and as much as 40% of alimentary glucose is taken up by the liver, and alterations in hepatic function influence glucose flux [91]. Gene-activating drug therapy reducing blood glucose induces hepatic glucokinase [23] which has been considered as a potential key target for glucose lowering therapy. A recent study showing that restoration of hepatic glucokinase expression beneficially corrects hepatic glucose flux and normalizes plasma glucose in diabetic fatty rats, supports this possibility [91]. 

The NRs are ligand-activated transcription factors that control a wide variety of metabolic processes by regulating the expression of genes encoding enzymes, transporters and other proteins involved in metabolic homeostasis [92]. NRs with known physiological ligands include endocrine receptors and adopted and enigmatic orphan receptors [92]. Ligands for endocrine NRs include glucocorticoid, mineralocorticoid, androgen, estrogen, thyroid hormone, retinoid acid and vitamin D. Table **1** presents ligands for orphan NRs with favorable metabolic effects. 

Large population studies indicate that absence in middle age of modifiable risk factors such as overweight, hyperglycemia, dyslipidemia and hypertension, are positively related to exceptional survival in old age [98, 99]. The reviewed studies indicate that these risk factors typical of metabolic syndrome are linked with the disturbed hepatic ER function affecting the fate of lipids, proteins, glucose and insulin.The studies also show that gene-activating therapy can eliminate abnormalities in the syndrome. It eliminates liver fat and reduces elevated plasma triglycerides and raises low HDL-C. The therapy can also normalize glucose tolerance and insulin level, promote weight control and reduce blood pressure. Healthy lifestyle choices together with natural gene activation have an important role in normalization of metabolic abnormalities, weight control and elimination of obesity, and decreasing blood pressure. 

HDL has anti-inflammatory, antioxidant, antiapoptopic, neuroprotective, vasodilatory and anti-thrombotic effects that protect cells from the effects of aging process [51]. Plasma HDL levels decline with age in prospective studies but cross-sectionally, by contrast, they do not change much or even slightly increase with age suggesting that people with still high HDL levels survive [51, 52]. In very old people age correlates positively with HDL2, a low HDL is very rare among them [51], and high HDL-C and apo AI correlate with cognitive function in advanced age [64]. 

A number of factors and activities can prevent cognitive impairment in man. Elimination of modifiable reasons of obesity, DM, dyslipidemia, heart disease, stroke, and hypertension, delays cognitive decline and development of dementia [11, 100]. Active participation in stimulating cognitive, social, and physical activities, and healthy diet, also have a positive delaying effect on the onset of dementia [11, 100]. Studies showing that carotid intimal thickness predicts accelerated cognitive decline among adults without clinical vascular disease [101], and that atherosclerosis in intracerebral arteries is an independent risk factor for dementia [102], indicate that activities and therapies protecting arteries from atherosclerosis are central for maintaining cognitive health. 

## CONCLUSIONS

Both endogenous and exogenous factors **i**ncluding living habits influence life maintaining functions. They activate genes in the processes that beneficially affect the fate of lipids, proteins and glucose, and protect cells from the effects of aging process. Gene-activating compounds induce reparation of altered cellular structures consequent to ER stress, and normalize and improve metabolic functions. They prevent and regress atherosclerosis and, by restoring normal glucose-insulin homeostasis, remove metabolic syndrome and DM2. The gene-activation produces positive changes that eliminate major cardiovascular and metabolic diseases, prevent cognitive impairment and dementia, and promote well-being and active living in advanced age. 

## Figures and Tables

**Table 1:** Orphan NRs and their Ligands with Effects on Metabolic Disorders/Diseases

Receptor	Endogenous ligand	Exogenous ligand	Disorder/Disease	References
LXR	Oxysterols, bile acids	TO901317	Dyslipidemia, atherosclerosis	[27, 92, 93]
LXR	Glucose	GW3965	Hyperglycemia	[21, 94]
PPARα	Fatty acids	Fibrate	Dyslipidemia, atherosclerosis	[92]
PPARγ	Fatty acids	Glitazone	DM, glucose intolerance, insulin resistance	[92]
PPAR α – γ		Statin	Dyslipidemia, atherosclerosis	[95]
PPARδ	Fatty acids	GW501516	Dyslipidemia, atherosclerosis	[92]
PXR	Bile acids	Rifampicin	Cholestasis, jaundice	[92, 96]
PXR-CAR		Phenobarbital	DM2, glucose intolerance, insulin resistance	[7, 8]
PXR-CAR		Phenobarbital	Hepatic steatosis	[7, 20]
PXR-CAR		Phenobarbital	Hyperbilirubinemia, cholestasis	[93]
CAR	Steroids, Bile acids	CITCO	Cholestasis, jaundice	[92, 93]
CAR		TCPOBOP	Glucose intolerance, insulin resistance, fatty liver	[83, 84]
FXR	Bile acids	Chenodeoxycholic acid	Cholestasis	[92]
FXR		GW4046	Dyslipidemia, hyperglycemia, insulin resistance	[97]

## ABBREVIATIONS

ABC: = ATP-binding cassette
AD: = Alzheimer`s disease
AP: = antipyrine
apo: = apolipoprotein
CAR: = constitutive androstane receptor
CCTα: = CTP:phosphocholine cytidylyltransferase-α
CHD: = coronary heart disease
CYP: = cytochrome
ER: = endoplasmic reticulum
FXR: = farnesoid X receptor
HMGCoAR: = hydroxymethylglutaryl-coenzyme A reductase
IRI: = immunoreactive insulin
LCAT: = lecithin:cholesterol acyltransferase
LDLR: = low density lipoprotein receptor
LXR: = liver X receptor
PB: = phenobarbital
PC: = phosphatidylcholine
PPAR: = peroxisome proliferator-activated receptor
PXR: = pregnane X receptor
SIRT1: = sirtuin 1
T-C: = total cholesterol

## References

[1] Bonow RO, Smaha LA, Smith SC Jr, Mensah GA, Lenfant C (2002). The international burden of cardiovascular disease: responding to the emerging global epidemic. Circulation.

[2] Brown MT, LeRoith D (2010). Overcoming challenges in type 2 diabetes management to improve patient outcomes. Expert Rev Endocrinol Metab.

[3] Cornier M-A, Dabelea D, Hernandez TL (2008). The metabolic syndrome. Endocr Revs.

[4] Grundy SM (2008). Metabolic syndrome pandemic. Arteriocler Thromb Vasc Biol.

[5] Luoma PV, Sotaniemi EA, Pelkonen RO, Ehnholm C, Pilli-Sihvola AS, Laaksovirta T (1980). Plasma high density lipoprotein and liver microsomal enzyme activity in man. The Medical Research Council 1977- 1979.

[6] Luoma PV (1997). Gene activation, apolipoprotein A-I/ high density lipoprotein, atherosclerosis prevention and longevity. Pharmacol Toxicol.

[7] Sotaniemi EA, Arranto AJ, Sutinen S, Stengård JH, Sutinen Sirkka (1983). Treatment of noninsulin-dependent diabetes mellitus with enzyme inducers. Clin Pharmacol Ther.

[8] Lahtela JT, Arranto AJ, Sotaniemi EA (1985). Enzyme inducers improve insulin sensitivity in non-insulin-dependent diabetic subjects. Diabetes.

[9] Zelcer N, Khanlou N, Clare R (2007). Attenuation of neuroinflam-mation and Alzheimer`s disease pathology by liver X receptors. PNAS.

[10] Koldamova R, Fitz NF, Lefterov I (2010). The role of ATP-binding cassette transporter A1 in Alzheimer`s disease and neurodegeneration. Biochim Biophys Acta.

[11] Ritchie K, Carrière I, Ritchie CW (2010). Designing prevention programmes to reduce of dementia: prospective cohort study of modifiable risk factors. BMJ.

[12] Luoma PV, Sotaniemi EA, Pelkonen RO (1987). Enzyme induction, lipids and apolipoproteins. Enzyme Induction in Man.

[13] Mandl J, Mèszàros T, Bànhegyi G, Hunyadu L, Csala M (2009). Endoplasmic reticulum: nutrient sensor in physiology and pathology. Trends Endocr Metab.

[14] Remmer H (1967). Die Induktion arzneimittelabbauender Enzyme im endoplasmatischen Retikulum der Leberzelle durch Pharmaka. Dtsch Med Wochenschr.

[15] Orrenius S (1965). Further studies on the induction of the drug-hydroxylating enzyme system of liver microsomes. J Cell Biol.

[16] Conney AH (1967). Pharmacological implications of microsomal enzyme induction. Pharmacol Revs.

[17] Luoma PV, Savolainen MJ, Sotaniemi EA, Pelkonen RO, Arranto AJ, Ehnholm C (1983). Plasma high density lipoprotein and liver lipids and proteins in man. Relation to hepatic histology and microsomal enzyme induction. Acta Med Scand.

[18] Savolainen MJ, Arranto AJ, Hassinen IE, Luoma PV, Pelkonen RO, Sotaniemi EA (1985). Relationship between lipid composition and drug metabolizing capacity of human liver. Eur J Clin Pharmacol.

[19] Luoma PV, Savolainen MJ, Sotaniemi EA, Arranto AJ, Pelkonen RO (1985). Plasma HDL cholesterol and glucose in non-insulin-dependent diabetics related to liver lipids and micromal enzyme activity. Acta Med Scand.

[20] Karvonen I, Stengård JH, Huupponen R, Stenbäck FG, Sotaniemi EA (1989). Effects of enzyme induction therapy on glucose and drug metabolism in obese mice model of non-insulin dependent diabetes mellitus. Diabetes Res.

[21] Mitro N, Mak PA, Vargas L (2007). The nuclear receptor LXR is a glucose sensor. Nature.

[22] Orrenius S, Ericsson LE (1966). On the relationship of liver glucose- 6 -phosphatase to the proliferation of endoplasmic reticulum in Phenobarbital induction. J Cell Biol.

[23] Karvonen I, Stengård JH, Saarni HU, Stenbäck F, Sotaniemi EA (1987). Hepatic mixed function oxidase system and enzymatic glucose metabolism in rats. Diabetes Res.

[24] Kontush A, Chapman MJ (2006). Functionally defective high-density lipoprotein: a new therapeutic target at the crossroads of dyslipidemia, inflammation, and atherosclerosis. Pharmacol Revs.

[25] Hoofnagle AN, Vaisar T, Mitra P (2010). HDL lipids and insulin resistance. Curr Diab Rep.

[26] Tchoua U, Gillard BK, Pownall HJ (2010). HDL superphospholipidation enhances key steps in reverse cholesterol transport. Atherosclerosis.

[27] Tontonoz P, Mangelsdorf DJ (2003). Liver X receptor signaling pathways in cardiovascular disease. Mol Endocrinol.

[28] Brewer BH Jr (2007). HDL metabolism and the role of HDL in the treatment of high-risk patients with cardiovascular disease. Curr Cardiol Rep.

[29] Zarubica A, Trompier D, Chimini G (2007). ABCA1, from pathology to membrane function. Pflugers Arch – Eur J Physiol.

[30] Björkhem I, Diczfalusy U (2002). Oxysterols, friends, foes, or just fellow passengers. Arterioscler Thromb Vasc Biol.

[31] Luoma PV (2007). Cytochrome P450 - physiological key factor against cholesterol accumulation and the atherosclerotic vascular process. Ann Med.

[32] Ross R (1999). Atherosclerosis–an inflammatory disease. New Engl J Med.

[33] Navab M, Ananthramaiah GM, Reddy ST (2004). The oxidation hypothesis of atherogenesis; the role of oxidized phospholipids and HDL. J Lipid Res.

[34] Joseph SB, Castrillo A, Laffitte BA, Mangelsdorf DJ, Tontonoz P (2003). Reciprocal regulation of inflammation and lipid metabolism by liver X receptors. Nature Med.

[35] Mashima R, Yamamoto Y, Yoshimura S (1998). Reduction of phosphatidylcholine hydroperoxide by apolipoprotein A-I: purification of the hydroperoxide-reducing proteins from human blood. J Lipid Res.

[36] Yamamoto Y, Nagata Y, Niki E, Watanabe K, Yoshimura S (1993). Plasma glutathione peroxidase reduces phosphatidylcholine hydroperoxide. Biochem Biophys Res Commun.

[37] Bisoendial RJ, Hovingh K, Levels JHM (2003). Restoration of endothelial function by increasing high-density lipoprotein in subjects with isolated low high-density lipoprotein. Circulation.

[38] Luoma PV, Rautio A, Stengård J, Sotaniemi EA, Marniemi J (1990). High-density lipoprotein subfractions, apolipoproteins and antipyrine clearance in normal subjects. Eur J Clin Pharmacol.

[39] Xiao L, Xie X, Zhai Y (2010). Functional crosstalk of CAR-LXR and ROR-LXR in drug metabolism and lipid metabolism. Adv Drug Deliv Rev.

[40] Luoma PV, Sotaniemi EA, Arranto AJ (1983). Serum LDL cholesterol, the LDL/HDL cholesterol ratio and liver microsomal enzyme induction evaluated by antipyrine kinetics. Scand J Clin Lab Invest.

[41] Miller M, Burgan RG, Osterlund L, Segrest JP, Garber DW (1995). A prospective, randomized trial of phenytoin in nonepileptic subjects with reduced HDL cholesterol. Arterioscler Thromb Vasc Biol.

[42] Luoma PV, Sotaniemi EA, Pelkonen RO, Savolainen MJ, Ehnholm C (1982). Induction and lipoproteins. Lancet.

[43] Luoma PV, Arranto AJ, Ehnholm C, Sotaniemi EA (1981). Liver histological changes and plasma high density lipoproteins in man. Res Commun Chem Pathol Pharmacol.

[44] Jacobs RL, Lingrell S, Zhao Y, Francis GA, Vance DE (2008). Hepatic CTP:phosphocholine cytidylyltransferase-α is a critical predictor of plasma high density lipoprotein and very low density lipoprotein. J Biol Chem.

[45] Gehrig K, Lagace TA, Ridgway ND (2009). Oxysterol activation of phosphatidylcholine synthesis involves CTP:phosphocholine cytidylyltransferase α translocation to the nucleus. Biochem J.

[46] Mäkelä SM, Jauhiainen M, Ala-Korpela M (2008). HDL2 of heavy alcohol drinkers enhances cholesterol efflux from raw macrophages *via* phospholipid- rich HDL2b particles. Alcoholism: Clin Exper Res.

[47] Gelissen IC, Harris M, Rye KA (2006). ABCA1 and ABCG1 synergize to mediate cholesterol export to apoA-I. Arterioscler Thromb Vasc Biol.

[48] Nanjee MN, Cooke CJ, Garvin R (2001). Intravenous apoA-I/lecithin discs increase pre-β-HDL concentration in tissue fluid and stimulate reverse cholesterol transport in humans. J Lipid Res.

[49] Hsia SL, Duncan R, Schob AH (2000). Serum levels of high-density lipoprotein phospholipids correlate with severity of angiographically defined coronary disease. Atherosclerosis.

[50] Luoma PV (2010). Gene activation regresses atherosclerosis, promotes health, and enhances longevity. Lipids in Health and Disease.

[51] Walter M (2009). Interrelationships among HDL metabolism, aging, and atherosclerosis. Arterioscler Thromb Vasc Biol.

[52] Arai Y, Hirose N (2004). Aging and HDL metabolism in elderly people more than 100 years old. J Atheroscler Thromb.

[53] Lahtela JT, Gachalyi B, Eksymä S, Hämäläinen A, Sotaniemi EA (1986). The effect of enzyme inducing and inhibiting drugs on insulin mediated glucose metabolism in man. Br J Clin Pharmacol.

[54] Lahtela JT, Arranto AJ, Stenbäck F, Sotaniemi EA (1986). Insulin-mediated glucose metabolism is related to liver structure and microsomal function. Scand J Gastroent.

[55] Laffitte BA, Mangelsdorf DJ, Tontonoz P (2003). Activation of liver X receptor improves glucose tolerance through coordinate regulation of glucose metabolism in liver and adipose tissue. PNAS.

[56] Luchsinger JA, Gustafson DR (2009). Adiposity, type 2 diabetes and Alzheimer`s disease. J Alzheimers Dis.

[57] Garner B (2010). Lipids and Alzheimer`s disease. Biochim Biophys Acta.

[58] Ricobaraza A, Cuadrado-Tejedor M, Pères-Mediavilla A, Frechilla D, Del Rio J, Garcia-Osta A (2009). Phenylbutyrate ameliorates cognitive deficit and reduces tay pathology in an Alzheimer`s disease mouse model. Neuropsychopharmacology.

[59] Wang I, Schuster GU, Hultenby K, Zhang Q, Andersson S, Gustafsson JA (2002). Lipid X receptors in the central nervous system: from lipid homeostasis to neuronal degeneration. PNAS.

[60] Hirsch-Reinshagen V, Burgess BL, Wellington CL (2009). Why lipids are important for Alzheimer disease. Mol Cell Biochem.

[61] Fan J, Donkin J, Wellington C (2009). Greasing the wheels of Aβ clearance in Alzheimer`s disease: The role of lipids and apolipoprotein E. Biofactors.

[62] Hartz AMS, Miller DS, Bauer B (2010). Restoring blood-brain barrier P-glycoprotein reduces brain amyloid-β in a mouse model of Alzheimer`s disease. Mol Pharmacol.

[63] Singh-Manoux A, Gimeno D, Kivimaki M, Brunner E, Marmot MG (2008). Low HDL cholesterol is a risk factor for deficit and decline in memory in midlife. Arterioscler Thromb Vasc Biol.

[64] Atzmon G, Gabriely I, Greiner W, Davidson D, Schechter B, Barzilai N (2002). Plasma HDL levels correlate with cognitive function in exceptional longevity. J Gerontol Med Sci.

[65] Craft S (2009). The role of metabolic disorders in Alzheimer disease and vascular dementia. Arch Neurol.

[66] Björkhem I (2006). Crossing the barrier: oxysterols as cholesterol transporters and metabolic modulators in the brain. J Intern Med.

[67] Papassotiropoulos A, Streffer JR, Tsolaki M (2003). Increased brain beta-amyloid load, phosphorylated tau, and risk of Alzheimer disease associated with intronic CYP46 polymorphism. Arch Neurol.

[68] Hotamisligil GS (2010). Endoplasmic reticulum stress and atherosclerosis. Nature Med.

[69] Özcan U, Cao Q, Yilmaz E (2004). Endoplasmic reticulum stress links obesity, insulin action, and type 2 diabetes. Science.

[70] Engin F, Hotamisligil GS (2010). Restoring endoplasmic reticulum function by chemical chaperones: an emerging therapeutic approach for metabolic disease. Diabetes Obes Metab.

[71] Schuck S, Prinz WA, Thorn KS, Voss C, Walter P (2009). Membrane expansion alleviates endoplasmic reticulum stress independently of the unfolded protein response. J Cell Biol.

[72] Sciburi R, Bommiasamy H, Buldak GL (2007). Coordinate regulation of phospholipid biosynthesis and secretory pathway gene expression in XBP-1 (S)-induced endoplasmic reticulum biogenesis. J Biol Chem.

[73] Erbay E, Babaev VR, Mayers JR (2009). Reducing endoplasmic reticulum stress through a macrophage lipid chaperone alleviates atherosclerosis. Nat Med.

[74] Gregor MF, Yang L, Fabbrini E (2009). Endoplasmic reticulum stress is reduced in tissues of obese subjects after weight loss. Diabetes.

[75] Özcan U, Ylimaz E, Özcan L (2006). Chemical chaperones reduce ER stress and restore glucose homeostais in a mouse model of type 2 diabetes. Science.

[76] Kars M, Yang L, Gregor MF (2010). Tauroursodeoxycholic acid may improve liver and muscle but not adipose tissue insulin sensitivity in obese men and women. Diabetes.

[77] Kim EJ, Um SJ (2008). SIRT1: roles in aging and cancer. BMB Reports.

[78] Rodgers JT, Puigserver P (2007). Fasting -dependent glucose and lipid metabolic response through hepatic sirtuin 1. PNAS.

[79] Feige JM, Auwerx J (2007). DisSirting on LXR and cholesterol metabolism. Cell Metab.

[80] Dullens SPJ, Plat J, Mensink RP (2007). Increasing apoA-I production as a target for CHD risk reduction. Nutrition Metab & Cardiovasc Dis.

[81] Lamon-Fava S, Diffenderfer MR, Barrett PHR (2008). Extended - release niacin alters the metabolism of plasma apoliprotein (apo) A-I and apo-B containing lipoproteins. Arterioscler Thromb Vasc Biol.

[82] Hofman MK, Weggemans RM, Zock PL, Schouten EG, Katan MB, Princen HMG (2004). CYP7A1 A-278C polymorphism affects the response of plasma lipids after dietary cholesterol or cafesterol interventions in humans. J Nutr.

[83] Gao J (2009). The constitutive androstane receptor is an anti-obesity nuclear receptor that improves insulin sensitivity. J Biol Chem.

[84] Dong B, Saha PK, Huang W (2009). Activation of nuclear receptor CAR ameliorates diabetes and fatty liver disease. PNAS.

[85] Stampfer MJ (2006). Cardiovascular disease and Alzheimer`s disease: common links. J Intern Med.

[86] Estruch R, Martinez-Gonzalez MA, Corella D (2006). Effects of a Mediterranien-style diet on cardiovascular risk factors. A randomized trial. Ann Intern Med.

[87] Scarmeas N, Luchsinger JA, Schupf N, Brickman AM, Cosentino S, Tang MX (2010). Physical activity, diet, and risk of Alzheimer disease. JAMA.

[88] Jimènez-Gomèz J, Marin C, Pèrez-Martinèz P (2010). A low-fat, high-complex carbohydrate diet supplemented with long-chain[n-3] fatty acids alters the postprandial lipoprotein profile in patients with metabolic syndrome. J Nutr.

[89] Pan XR, Li GW, Hu YH (1997). Effects of diet and exercise in preventing NIDDM in people with impaired glucose tolerance. Diab Care.

[90] Geda YE, Roberts RO, Knopman DS (2010). Physical exercise, aging, and mild cognitive impairment. Arch Neurol.

[91] Torres TP, Catlin RL, Chan R (2009). Restoration of hepatic glucokinase expression corrects hepatic glucose flux and normalizes plasma glucose in Zucker diabetic fatty rats. Diabetes.

[92] Sonoda J, Pei L, Evans RM (2008). Nuclear receptors: Decoding metabolic disease. FEBS Lett.

[93] Handschin C, Meyer UA (2005). Regulatory network of lipid-sensing nuclear receptors: roles of CAR, PXR, LXR, and FXR. Arch Biochem Biophys.

[94] Commerford RS, Vargas L, Dorfman SE (2007). Dissection of the insulin-sensitizing effect of liver X receptor ligands. Mol Endocrinol.

[95] Paumelle R, Staels B (2007). Peroxisome prolifertor-activated receptors mediate pleiotrophic actions of statins. Circ Res.

[96] Kliewer SA (2005). Cholesterol detoxification by nuclear pregnane X receptor. PNAS.

[97] Claudel T, Staels B, Kuipers F (2005). The farnesoid X receptor – a molecular link between bile acid and lipid and glucose metabolism. Arterioscler Thromb Vasc Biol.

[98] Sierra F, Hadley E, Suzman R, Hodes R (2009). Prospects of life span extension. Annu Rev Med.

[99] Willcox BJ, He Q, Chen R (2006). Midlife risk factors and healthy survival in man. JAMA.

[100] Middleton LE, Yaffe K (2009). Promising strategies for the prevention of dementia. Arch Neurol.

[101] Wendell CR, Zonderman AB, Metter JM, Najjar SS, Wadstein SR (2009). Carotid intimal medial thickness predicts cognitive decline among adults without clinical vascular disease. Stroke.

[102] Dolan H, Crain B, Troncoso J, Resnick SM, Zonderman AB, Obrien RJ (2010). Atherosclerosis, dementia, and Alzheimer disease in the Baltimore longitudinal study of aging cohort. Ann Neurol.

